# Inhibitory effects of *Clostridium butyricum* culture and supernatant on inflammatory colorectal cancer in mice

**DOI:** 10.3389/fimmu.2023.1004756

**Published:** 2023-04-04

**Authors:** Wenfeng Pu, Hong Zhang, Tao Zhang, Xiaoguang Guo, Xiaoqing Wang, Shaohui Tang

**Affiliations:** ^1^Department of Gastroenterology, The First Affiliated Hospital, Jinan University, Guangzhou, Guangdong, China; ^2^Department of Gastroenterology, Nan Chong Central Hospital, the Second Affiliated Hospital of North Sichuan Medical College, Sichuan, Nanchong, China; ^3^Department of Gastroenterology, Affiliated Hospital of North Sichuan Medical College, Sichuan, Nanchong, China; ^4^Department of Gastroenterology, West China School of Medicine, West China Hospital, Sichuan University, Chengdu, China; ^5^Department of Pathology, Nan Chong Central Hospital, the Second Affiliated Hospital of North Sichuan Medical College, Sichuan, Nanchong, China; ^6^Department of Nucler Medicine, Nan Chong Central Hospital, the Second Affiliated Hospital of North Sichuan Medical College, Sichuan, Nanchong, China

**Keywords:** colorectal cancer, *Clostridium butyricum*, colitis-associated cancer, gut microbiota, butyrate

## Abstract

*Clostridium butyricum* (CB) is a spore-forming, gram-positive and obligate anaerobic rod bacterium. CB can modulate the composition of the gut microbiome and promote the growth of beneficial microbes in the intestine by generating short-chain fatty acids (SCFAs), which in turn protect against colitis and prevents the formation of inflammatory-associated colorectal cancer (CRC) by ameliorating colon inflammatory processes. Yet, it remains unclear whether the culture and supernatant of CB could directly influence inflammatory CRC in mice. In this study, azoxymethane (AOM)+dextran sodium sulphate (DSS) was used to induce CRC model in C57BL/6 mice. Next, the serum levels of inflammatory cytokines, including interleukin-6 (IL-6), interleukin-10 (IL-10), and cytokines TNF-α, were measured and the pathohistological examination of the large intestine was performed. Both CB culture and supernatant were found to have anti-inflammatory properties. Subsequently, Western blot and Real-Time Quantitative PCR (RT-qPCR) revealed that CB and supernatant regulate the NF-κB/p65 pathway to inhibit the development and progression of inflammatory CRC in AOM+DSS-treated mice, which could be due to the high levels of butyric acid in the supernatant.

## 1 Introduction

Colorectal cancer (CRC) ranks third among all malignant tumors and is the fourth leading cause of cancer mortality worldwide. In the United States in 2020, there were about 147,950 new patients diagnosed with CRC diagnosed, 53,200 of whom died from the disease, including 17,930 cases and 3,640 deaths among those aged < 50 years old (1). According to the etiology, approximately 20% of CRC patients have a family history of CRC (2), while the others are sporadic CRC. Epidemiological studies have also demonstrated that the development and progression of CRC are multifactor and multistep processes influenced by environmental and genetic factors (3). Inflammation is associated with the progression of tumors (4), and about 20% of cancers are the direct outcomes of chronic inflammation (5). Long-term chronic and recurrent bowel inflammation is a risk factor for developing colitis-associated cancer (CAC). A previous study showed that the incidence of CAC among those suffering from inflammatory bowel disease (IBD) for 10 years, 20 years, and 30 years is 1.6%, 8.3%, and 18.4%, respectively (6).

Many studies have shown that microbiota interventions, such as probiotics and fecal microbiota transplants, can regulate the gut microbiota of hosts, and improve the barrier functions of the intestine, consequently leading to positive treatment effects (7–10). Some studies have shown that using probiotics to change gut microbiota is a potentially effective strategy to prevent and treat CRC (11, 12), while others have suggested that the alteration of gut probiotics is associated with CRC (13–15).

Compared to healthy individuals, CRC patients have a decreased amount of butyrate-producing microbes (16–20). *Clostridium butyricum* (CB) is a butyrate-producing, spore-forming anaerobic gram-positive, and obligate anaerobic rod bacterium. Butyrate producing bacteria could potentially be used for CRC treatment due to decreased content in the fecal matter of CRC patients (16, 21). In 1933, Dr. Chikaji Miyairi first isolated Strain MIYAIRI 588 from the feces of a healthy human. CB may modulate the composition of the gut microbiome and promote the growth of beneficial microbes in the intestine, such as Bifidobacterium and Lactobacillus (12–15). The fermentation of dietary fibers produces short-chain fatty acids (SCFAs) by intestinal microbiota (22). CB has fermentative properties and can consume undigested dietary fibers and generate SCFAs (23), which consist of 1-6 carbons, where acetate, butyrate, and propionate are the most abundant (24, 25). SCFAs protect against DSS-induced colitis (26–28) and prevents the formation of inflammatory-associated CRC by ameliorating colon inflammatory processes and limiting the activation of NF-κB and the expression of COX-2 (27). Xiao et al. (29) induced CAC mice models using 2,4,6-trinitro-benzenesulfonic acid (TNBS) and AOM, showing that CB regulates the production of proinflammatory cytokines, such as tumor necrosis factor-alpha (TNF-α) and interleukin (IL)-12, through microRNA 200c. Moreover, Liu et al. (30) investigated the effects of freeze-dried powder of CB on gut microbiota and CAC in mice, finding that CB reduces the expression of TNF-α, IL-6, while COX-2 decreases the phosphorylation of NF-κB and the level of Bcl-2, and increases the expression of Bax. Yet, no studies assessed whether the culture and supernatant of CB could equally influence inflammatory CRC in mice.

This study aimed to explore the prevention and treatment effect and mechanism of CB culture and its supernatant on CRC from the aspects of regulating intestinal flora, inhibiting intestinal inflammatory response, promoting apoptosis, etc., so as to provide guidance for the prevention and treatment of CRC through the regulation of clinical intestinal microecology.

## 2 Materials and methods

### 2.1 Mice

A total of 24 8–10 weeks old male C57BL/6 mice, weighing 23-24 g were purchased from the Beijing Huafukang Biotechnology Co., Ltd (License No.: SCK(Su)2018-000) and maintained in the specific pathogen-free animal laboratory of the Experimental Animal Center, North Sichuan Medical College (License No. of experimental animal facility: SYXK (Chuan) 2018-076) (NSMC ethical animal audit (2021) No.75). After acclimation for 1 week, the animals were randomly divided into four following groups: control (treated by AOM+DSS), bacteria (treated by AOM+DSS+CB culture), supernatant of bacteria (treated by AOM+DSS+CB supernatant), blank (treated by sterile drinking water).

### 2.2 Induction of mice models of CRC

**Figure 1:**
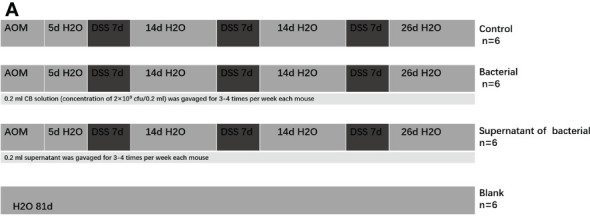
The mice models of CRC were induced by AOM+DSS and gavaged of CB culture and supernatant as intervention.

The mice models of CRC were induced as previously described (31). The mice in the blank group had free access to sterile drinking water and were intraperitoneally injected with 0.2 ml of sterilized saline for 5 days. The other three groups were intraperitoneally injected 10 mg/kg AOM (prepared in dark) and after 5 days, they were given 2% DSS for drinking without restriction. The total time of the treatment of mice was 81 days (**Figure 1A**).

### 2.3 Estimation of disease activity index and body weight

Water and food intake, hair conditions, runny or bloody stool, activities, and diet were assessed daily. The DAI was assessed based on the changes in body weight (measured once per week), fecal property, and hematochezia. It was calculated according to the criteria developed by OkayasuI (32) as follows: *DAI=(body weight score+fecal property score+hematochezia score)/3*. It was used to assess the clinical manifestations of mice by two investigators using the blinding method.

### 2.4 Preparation of CB culture and supernatant

A total of 250 g of reinforced clostridium medium (bio-108590) was purchased from Beijing Biobw Biotechnology Co; te medium contained (10 g of tryptone, 10 g of beef extract, 5 g of glucose, 5 g of sodium chloride, 3 g of yeast, 3 g of sodium acetate, 1 g of soluble starch, 0.5 g of L-cysteine hydrochloride and 15 g of agar). Briefly, 1000 ml ddH_2_O was added with 15 g agar and 50 g reinforced clostridium medium for high-pressure steam sterilization for 30 min. The medium was left to cool to 50°C. Then, 32-40 ml medium was placed in a 10 cm diameter plate. The top cover of the plate was slightly opened and dried in a biosafety cabinet at 37°C for 30 min. The dried plate was wrapped and stored in the refrigerator at 4°C for later use.

CB strain (ATCC19398) was purchased from the Beijing Biobw Biotechnology Co., Ltd and cultured on enhanced solid agar medium for reinforced clostridium medium at 37°C for 48 h. The bacterial solution was prepared at A600 = 0.5 and gradually diluted. 0.1 ml bacterial solution was inoculated on the reinforced clostridium medium plate and cultured in an anaerobic incubator at 37°C for 15 hours. The number of colonies in each culture dish was recorded, and the total number of colonies/mL of the original sample was calculated according to the dilution ratio.

To prepare the CB culture and supernatant, the bacteria were collected at optical density (OD)600 = 0.5 by centrifugation and re-suspended in sterile phosphate-buffered saline (PBS) to the required concentration for the experiments (2×10^9^ CFU/0.2 mL) (33) and then centrifuged (5000× g for 10 min at room temperature). The supernatant was then discarded, and the pellet resuspended with reinforced clostridium medium broth of equal volume. CB cultured in an anaerobic incubator at 37°C for 24 hour. After 24 h, the culture broth was centrifuged (5000× g for 10 min at room temperature).The supernatant was collected and filtered through a 0.22-µm filter (34). For the mice in the bacteria group, 0.2 mL CB culture (concentration of 2×10^9^ CFU/0.2 mL) was gavaged 3-4 times/week/mouse. For the supernatant group, 0.2 mL supernatant was gavaged 3-4 times/week/mouse. For the control groups, 0.2 mL PBS was gavaged 3-4 times/week/mouse.

### 2.5 Serum collection of the mice

Mice were anesthetized by inhalation with 4% isoflurane for 2 min, after which the blood was collected from the posterior orbital vein and allowed to clot naturally for 10–20 min, followed by centrifugation at 2000–3000 rpm for 10 min at room temperature (35, 36). The supernatant was stored at −80°C until further use.

### 2.6 Collection of intestinal tissues

After euthanasia, the mice were soaked in 75% alcohol for 5 min. Sterile instruments take samples from clean bench. Cleaning the large intestine with sterilized saline. After flattening, the tissues were fixed in 10% buffer formalin for 24 h, followed by trimming, dehydration, clearing, paraffin embedding, slicing, and hematoxylin-eosin staining. The residual intestinal tumor tissues were classified as small (<1 mm), moderate (1-2 mm), and large (>2 mm) tissues. RNA was extracted from part of colon tumor tissue by TRIZOL method. Intestinal tumor tissue was digested with 1 ml Trizol and centrifuged at 6500 rpm for 15 s for 3 times. Fully homogenized tissues were allowed to stand at room temperature for 5 min. The samples were centrifuged at the maximum speeded instantaneously and the supernatant was collected and added into 0.2 ml chloroform. The mixture was mixed and reacted at room temperature for 3 min (pink and white) and then centrifuged at 4°C, 12000 *g* × 15 min. The supernatant was transferred into a new 1.5 ml EP tube, and mixed with 0.5 ml isopropyl alcohol gently. The tube was allowed to stand at room temperature for 10 min. Next, it was centrifuged at 4°C, 12000 g × 10 min. The supernatant was discarded, and the precipitate was mixed with 1 ml 75% ethanol, shaken, and washed. This was followed by centrifugation at 4°C, 7500 g × 5 min. The supernatant was discarded whereas the precipitate was collected. The RNA was dissolved in 20 μl DEPC water in time. They were stored at −80°C until further use.

### 2.7 Microbiota analysis by 16S rRNA sequencing

#### 2.7.1 Fecal samples

The fecal samples were collected from 24 mice (6/group), frozen at −80°C, and then transferred on dry ice to the Beijing Novogene Biotechnology Co., Ltd for 16S rRNA sequencing.

#### 2.7.2 16S rRNA gene amplification and sequencing

##### 2.7.2.1 Extraction of genome DNA

About 1000 µl of the CTAB lysate was added into a 2.0 ml EP tube followed by addition of 20 µl lysozyme. An appropriate amount of the samples was added to the lysate and incubated at 65°C (for fecal samples the incubation was performed for 2 hours). The mixing ratio was reversed during the incubation to fully crack the sample. 950 µl of the supernatant was centrifuged and equal amount of phenol (pH 8.0): chloroform: isoamyl alcohol (25:24:1) was added and mixed by turning the tube upside down followed by centrifugation at 12000rpm for 10min. The supernatant was mixed with an equal volume of chloroform: isoamyl alcohol (24:1), mixed by turning the tube upside down. It was centrifuged at 12000 rpm for 10 min. The supernatant was added into a 1.5 mL centrifuge tube, followed by addition of 3/4 volume of isopropyl alcohol. The tube was shaken by mixing up and down, and allowed to settle at -20°C precipitation. It was centrifuged at 12000 rpm for 10 min and the liquid was poured out carefully not to pour out the precipitation. The precipitate was washed with 1ml 75% ethanol It was filtered 2 times, and the remaining small amount of liquid was centrifugal again to collect the precipitate by sucking out the supernatant with a pipette gun. The sample were blow-dried on an ultra-clean workbench or at room temperature (Avoid over-drying the DNA sample to be make it easier to dissolve). Subsequently, 51 µL ddH_2_O was added to dissolve the DNA sample and incubated at 55-60°C for 10min to accelerate the dissolution where necessary. 1 µl RNase A-digested RNA was added and then incubated at 37°C for 15min. DNA concentration and purity was monitored on 1% agarose gels. According to the concentration, DNA was diluted to l µg/µL using sterile water.

##### 2.7.2.2 Amplicon generation

16S rRNA/18S rRNA/ITS genes of distinct regions (16S V4/16S V3/16S V3-V4/16S V4-V5, 18SV4/18S V9, ITS1/ITS2, Arc V4) were amplified used specific primer (e.g. 16S V4: 515F-806R, 18S V4: 528F-706R, 18S V9: 1380F-1510R, et.al) with the barcode (**Table 1**). All PCR reactions were carried out with 15 μL of Phusion High-Fidelity PCR Master Mix (New England Biolabs); 0.2 μM of forward and reverse primers, and about 10 ng template DNA. Thermal cycling consisted of initial denaturation at 98°C for 1 min, followed by 30 cycles of denaturation at 98°C for 10 s, annealing at 50°C for 30 s, and elongation at 72°C for 30 s. Finally, elongation was about 5 minutes at 72°C to fully extend the remaining single-stranded DNA.

**Table 1:** Primer sequences used for region Real-time quantitative -PCR and 16SV3+V4 amplification.

Primers	Sequence
NF-kBp65	Forward 5’- GGA GCA CAG ATA CCA CCA AGA −3’ Reverse 5’- CGG CAG TCC TTT CCT ACA AG −3’
Bcl-2	Forward 5’- GGT GAA CTG GGG GAG GAT TG −3’ Reverse 5’- GTG CCG GTT CAG GTA CTC AG −3’
Bax	Forward 5’- CCC CGA GAG GTC TTT TTC CG−3’ Reverse 5’- CCG GAG GAA GTC CAA TGT CC −3’
V3+V4	Forward 5’- CCT AYG GGR BGC ASC AG−3’ Reverse 5’- GGA CTA CNN GGG TAT CTA AT −3’

##### 2.7.2.3 PCR products quantification and qualification

Mix same volume of IX loading buffer (contained SYB green) with PCR products and operate electrophoresis on 2% agarose gel for detection. PCR products was mixed in equidensity ratios. Then, mixture PCR products was purified with Qiagen Gel Extraction Kit (Qiagen, Germany).

##### 2.7.2.4 Library preparation and sequencing

Sequencing libraries were generated using TruSeq DNA PCR-Free Sample Preparation Kit (Illumina, USA) following manufacturer’s recommendations and index codes were added. The library quality was assessed on the Qubit2.0 Fluorometer (Thermo Scientific) and Agilent Bioanalyzer 2100 system. At last, the library was sequenced on an Illumina NovaSeq platform and 250 bp paired-end reads were generated.

##### 2.7.2.5 Data analysis

Paired-end reads was assigned to samples based on their unique barcode and truncated by cutting off the barcode and primer sequence. Paired-end reads were merged using FLASH (VI.2.7, http://ccb.jhu.edu/software/FLASH/). Quality filtering on the raw tags were performed under specific filtering conditions to obtain the high-quality clean tag (37) according to the QIIME (V1.9.1, http://qiime.org/scripts/splitlibrariesfastq.html) quality controlled process. The tags were compared with the reference database (Silva database, https://www.arb-silva.de/) using UCHIME algorithm (UCHIME Algorithm, http://www.drive5.com/usearch/manual/uchime_algo.html) to detect chimera sequences, and then the chimera sequences removed (38). Then the Effective Tags were finally obtained. T-test, MetaStat, LEfSe, Anosim and MRPP were used for species composition and colony results of grouped samples.

### 2.8 Enzyme-linked immunosorbent assay

The ELISA kits purchased from the Wuhan FineTest Biotechnology Co., Ltd were used to measure the serum levels of IL-6, IL-10, and TNF-a, according to the manufacturer’s instructions. The serum samples were diluted in dilution buffer at a 1:1 ratio, and three measurements were acquired for each sample. Subsequently, standards of different concentrations were prepared, and 100 μL was added to the corresponding wells. The plate was covered and then incubated at 37°C for 1.5 h. The cover was removed, and the plate was washed 2 times with wash buffer, after which 100 μL biotinylated antibody was added to each well and incubated at 37°C for 1 h. Subsequently, the plate was washed 3 times with wash buffer, and 100 μL SABC working solution was added at 37°C for 0.5 h. The plate was washed 5 times, followed by 90 μL TMB substrate solution in the dark for 15 min. After the reaction turned blue, 50 μL stopping solution was added to the wells, and the OD450 was measured. The data were converted to pg/mL using the standard curve.

### 2.9 Western blot

The extraction process of histopin produced soluble protein and ensured that the protein was not degraded. Tissues from the colon tumor were lysed in RIPA buffer containing a mixture of protease inhibitors (Beyotime, China). BCA assay was used to measure the protein concentration. After heat denaturation at 99°C, 30 µg of protein samples from each group were separated using 10-12% SDS-PAGE gels and were transferred to PVDF membranes (Millipore, USA). Each membrane was then blocked with 5% skim milk or 5% BSA for 2 h at room temperature, after which it was incubated overnight at 4°C with the following primary antibody: anti rabbit-NF-KB65 (CST, USA) (1:1000), antirabbit -Bcl-2 (CST, USA) (1:1000), antirabbit -Bax (abcam, UK) (1:1000). After washing with Tris-buffered saline (TBS) containing 0.1% Tween-20 (TBST), the membrane was incubated with horseradish peroxidase (HRP)-conjugated secondary antibody (Beyotime, China) for 1h at room temperature. The protein bands were detected using Immobilon^®^ Western Chemiluminescent HRP Substrate (Millipore, USA). The immunoreactive bands were analyzed by lmage for densitometrical quantifications.

### 2.10 Real-time quantitative polymerase chain reaction (RT-qPCR)

The primers for real-time qPCR were synthesized by Shanghai Sangon Biotech Co., Ltd; the sequences are listed in **Table 1**. Next, reverse transcription kit HiScript III RT SuperMix for qPCR (+gDNA wiper) (Batch No. R323-01; Size: 100 rxn) was used for cDNA synthesis according to the manufacturer’s instructions, after which PCR was performed using ChamQ Universal SYBR qPCR Master Mix qPCR (Batch No. Q711; Size: 25 mL), according to the manufacturer’s protocol. The reaction system was as follows: 10 μL SYBR Green Supermix, 1 μL forward primer (10 μmol/L), 1 μL backward primer (10 μmol/L), 6 μL deionized water, and 2 μL cDNA template (50 ng/μL). The reaction conditions were: initial denaturation at 95°C for 30 s, followed by 40 cycles of denaturation at 95°C for 3 s, and annealing and extension at 60°C for 30 s each. The melting curve was acquired by denaturation at 95°C for 15 s to ensure a single peak in the melting curve of the amplification products, and the CT value was within the confidential range.

### 2.11 Statistical analysis

GraphPad Prism software (GraphPad Prism 8) was used for all statistical analyses. The data were expressed as mean ± standard error of the mean (SEM). The data were statistically analyzed by one-way or two-way ANOVA. A P-value <0.05 represented a statistically significant difference.

## 3 Results

### 3.1 CB culture and supernatant altered the gut microbiota in mice

#### 3.1.1 Gut microbiota differed between groups

**Figure 2:**
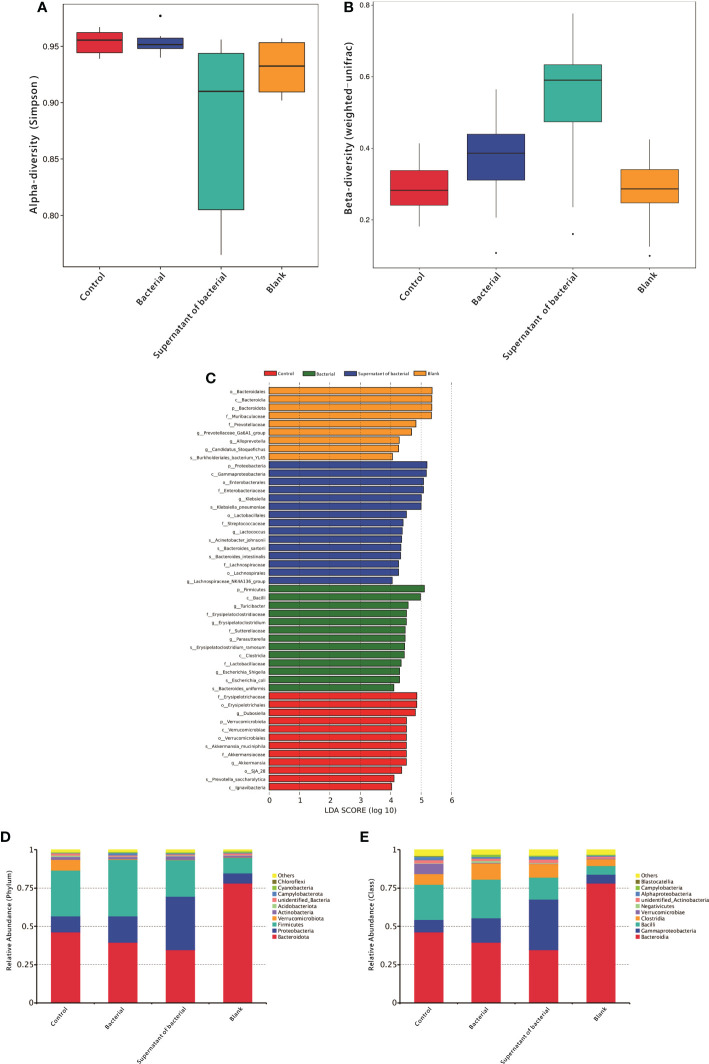
Analysis of gut microbiota in mice. **(A)** Alpha-diversity using the Simpson scale to measure the species richness and evenness. **(B)** Blpha-diversity using weighted_unifrac scale to measure distribution of different sequences. **(C)** LDA of gut microbiota among different groups. **(D)** Measuring the abundance of dominant microflora among different groups at the phylum level. **(E)** Measuring the abundance of the dominant microflora among different groups at the class level.

A 16S rRNA sequencing analysis investigated the differences in gut microbiota derived from four treatments. First, the alpha diversity was measured using the Simpson scale. Most of the groups manifested similar tendencies; however, the control group harbored microbiota with a high alpha diversity compared to the supernatant of the bacteria group (P = 0.04) (**Figure 2A**). Then beta-diversity was measured using weighted_unifrac scale. Again, there were significant differences between the control group and bacteria and the supernatant of the bacteria group (P = 0.02 and P = 0.00, respectively) (**Figure 2B**).

#### 3.1.2 Differences in the predominance of bacterial communities

In order to detect the differences in the predominance of bacterial communities between these groups, high-dimensional class comparisons were performed using the linear discriminant analysis (LDA) of effect size (LEfSe) (LDA score > 4.0, P < 0.05) (**Figure 2C**). The control group was found to have high f-Erysipelotrichaceae, o-Erysipelotrichales, g-Dubosiella, p-Verrucomicrobiota. In the bacterial group, p-Firmicutes, c-Bacilli, g-Turicibacter, and f-Erysipelatoclostridium were abundant. The supernatant group had a high content of p-Proteobacteria c-Gammaproteobacteria, o-Enterobacterales, and f-Enterobacteriaceae

#### 3.1.3 Landscape of the gut microbiota in all the available samples

At the phylum level, the content of Proteobacteria in the two groups increased from 10.3% and 6.5% to 17.0% and 34.9%, respectively, after the administration of bacteria and supernatant (**Figure 2D**). At the class level, the content of Gammaproteobacteria in the two groups increased from 8.1% and 5.9% to 15.9.0% and 32.8%, respectively, after the administration of bacteria and supernatant (**Figure 2E**).

### 3.2 CB culture and supernatant reduced DAI in mice

**Figure 3:**
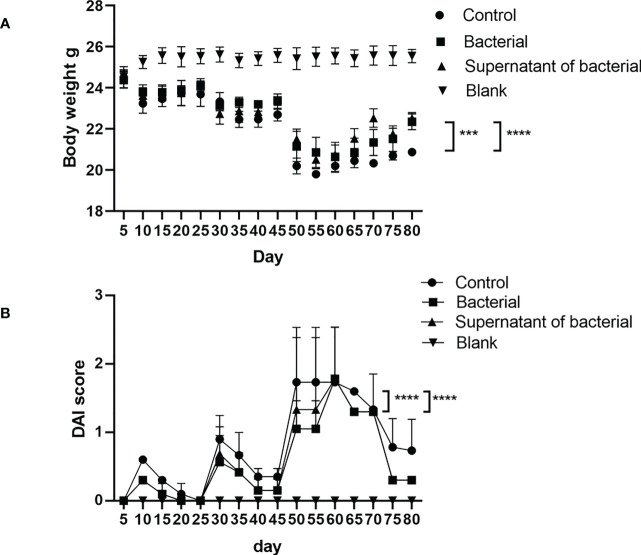
Analysis of body weight and DAI in mice. **(A)** Comparison of body weight among the different groups. **(B)** Comparison of DAI among different groups. The data are presented as mean ± SEM and compared using two-way ANOVA. ***P<0.0005, and ****P<0.0001.

Body mass reduction, runny or even bloody stool, reduced activities, and diet reduction were observed in the control, bacteria, and supernatant groups at 2-3 days after model induction. However, the manifestations in the bacteria and supernatant groups were less severe than in the control group. On the five days of observation, the body weight in the four groups did not significantly differ (P > 0.05).On the last day of observation, the body weight in the bacteria and supernatant groups was significantly less reduced compared to the control group (P < 0.0005 and P < 0.0001) (**Figure 3A**). On the 65 days of observation, the DAI was significantly different in the bacteria and supernatant groups compared to the control group (P < 0.0001) (**Figure 3B**) but did not significantly differ between the bacteria and supernatant groups (P > 0.05).

### 3.3 CB culture and supernatant alleviate inflammation in the large intestine and CRC-induced by AOM/DSS

**Figure 4:**
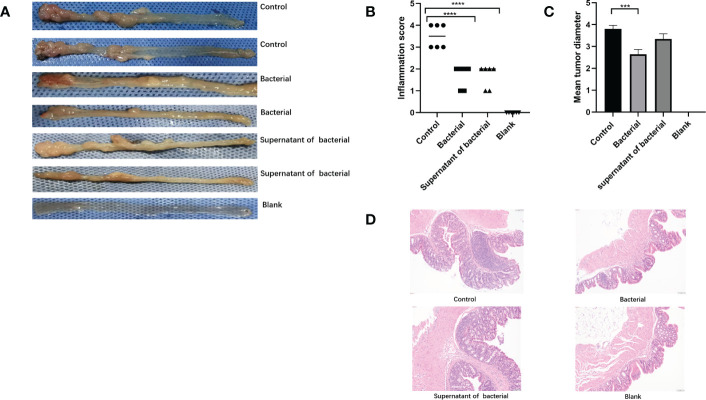
Induction of mice models of CRC and inflammatory scores. **(A)** Intestinal tumor tissues in the mice of the control, bacteria, supernatant, and blank groups. **(B)** Comparison of intestinal inflammatory score among different groups. **(C)** Comparison of tumor length among different groups. **(D)** HE findings among different groups. The data are described as mean ± SEM and compared using one-way ANOVA. ***P<0.0005, and ****P<0.0001.

The pathohistological scores of colon inflammation in mice were assessed according to the Neurath criteria (39) (**Figure 4A**). In the control group, inflammatory cell infiltration, disrupted structures, reduced number of goblet cells, and severe pathological damage of the colon were observed in the colonic mucosa, and the pathological score was significantly elevated. The inflammatory infiltration degree was lower in the bacteria and supernatant groups than in the control group. The pathological damages were less severe, and the pathological scores were also significantly lower in the bacteria and supernatant groups compared to the control group (P < 0.0001 and P < 0.0001, respectively) (**Figures 4B, D**). We went through intestinall lesions tissue HE staining. The lesions were confirmed as tumors.The tumor formation rate of three groups was 100%. In addition, the lengths of tumors were significantly lower in the bacteria than in the control group (P < 0.0005) (**Figure 4C**).

### 3.4 CB culture and supernatant alter the release of inflammatory cytokines

**Figure 5:**
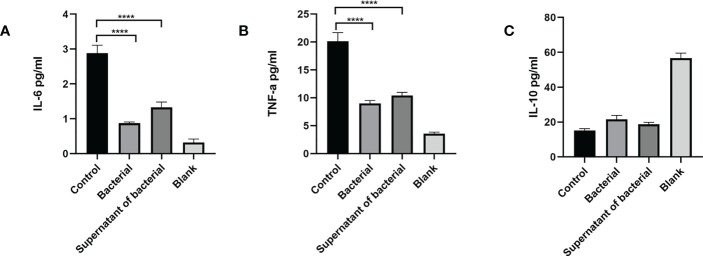
CB culture and supernatant inhibit the expression of pro-inflammatory cytokines (IL-6,TNF-α) and promote the expression of anti-inflammatory cytokines (IL-10), as shown by ELISA. **(A)** Serum level of inflammatory cytokine IL-6. **(B)** Serum level of inflammatory cytokine TNF-α. **(C)** Serum level of inflammatory cytokine IL-10. Data are presented by mean ± SEM and compared using one-way ANOVA. ****P<0.0001.

ELISA was used to measure the levels of inflammatory cytokines in peripheral serum. The findings showed that the IL-6 level was significantly higher in the control group compared to the bacteria and supernatant groups (P < 0.0001) (**Figure 5A**). The level of TNF-α was significantly higher in the control group compared to the bacteria and supernatant groups (P < 0.0001) (**Figure 5B**). The IL-10 level was lower in the control group compared to bacteria and supernatant groups (**Figure 5C**).

### 3.5 CB bacteria and supernatant ameliorate CAC by inhibiting the NF-κB pathway and the release of NF-κB and Bcl-2, and promoting the release of Bax

**Figure 6:**
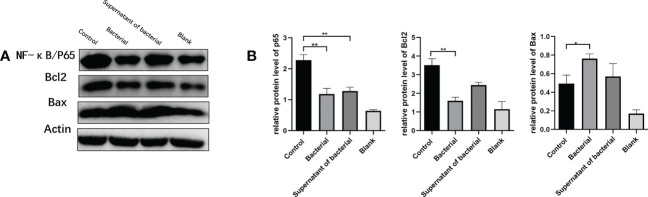
CB culture and supernatant inhibited the activation of the NF-κB pathway. **(A)** Representative Western blot results of NF-κB/P65, Bcl-2, and Bax. **(B)** Comparison of the expressions of NF-κB/P65, Bcl-2, and Bax proteins. The data are represented by mean ± SEM and compared using one-way ANOVA. *P<0.05, **P<0.001.

The nuclear transcription factor NF-κB has a major role in inflammatory and autoimmune diseases. To explore whether CB culture and supernatant regulate the NF-κB pathway to exert the anti-inflammation and pro-apoptosis effects, the levels of the representative components of this cascade, NF-κB/P65, Bcl-2, and Bax, were investigated. Our findings showed that the levels of NF-κB and Bcl-2 were higher, while the Bax level was lower in the control group than in the bacteria and supernatant groups (**Figures 6A, B**), which indicated that both CB culture and supernatant promote the pro-apoptosis gene expression and inhibit the anti-apoptosis gene expression, thus inhibiting the development and progression of CRC.

### 3.6 Measuring the transcription factor NF-κB, Bcl-2, and Bax in the colon tissues by RT-qPCR

**Figure 7:**
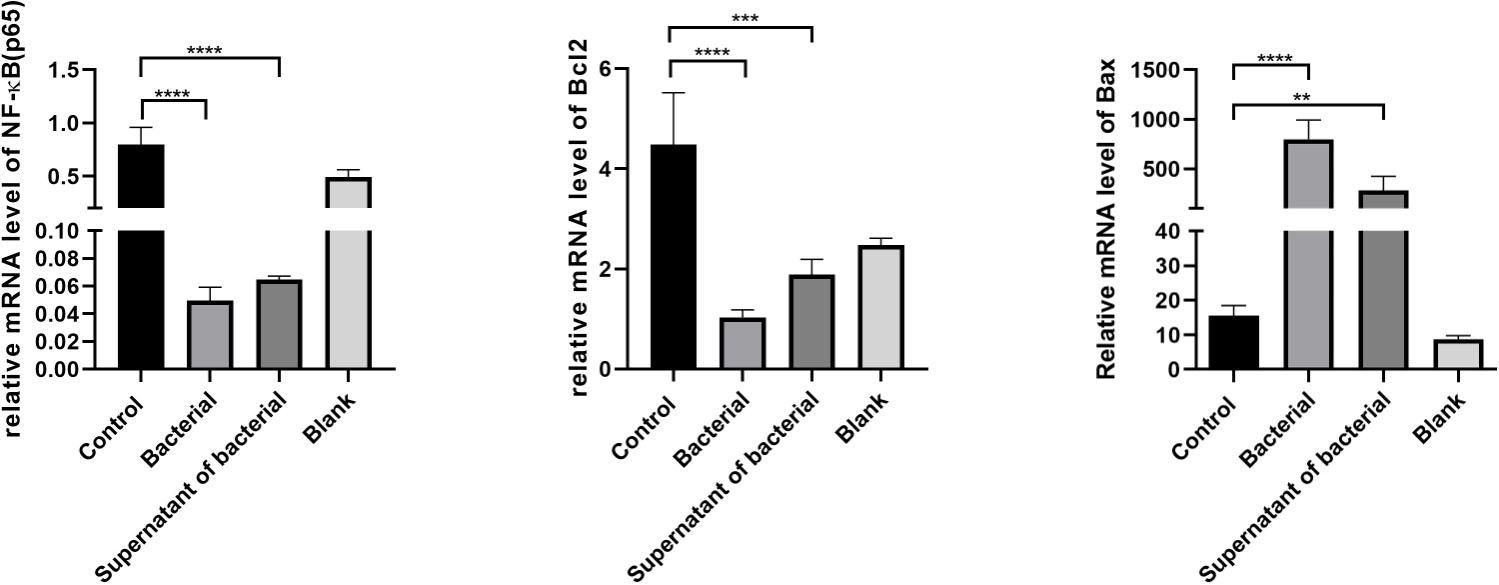
The expression of NF-κB, Bcl-2, and Bax in colon tissues by RT-qPCR. The data are described by mean ± SEM and compared by one-way ANOVA. **P<0.001, ***P<0.0005, and ****P<0.0001.

RT-qPCR measured the expressions of NF-κB, Bcl-2, and Bax in colon tissues. Our results showed that the level of NF-κB was lower in the bacteria and supernatant groups compared to the control group. In addition, the Bcl-2 expression was significantly lower, while Bax expression was significantly higher in the bacteria and supernatant groups than in the control group (**Figure 7A**). Treatment by CB culture and supernatant reduced the expression of Bcl-2 and increased the expression of Bax, indicating that CB culture and supernatant promote the expression of pro-apoptosis gens and inhibit the expression of anti-apoptosis gene, thus inhibiting the development and progression of CRC.

## 4 Discussion

In the present study, we explored the effect of CB culture and supernatant on the treatment of CRC and the underlying mechanisms in relation to the regulation of gut microbiota, inhibition of intestinal inflammation, regulation of host immune responses, and anti-apoptosis in mice. Our results showed that both CB and supernatant could regulate the intestinal microecology and exert beneficial effects in treating CRC.

CB preparations have been used in clinical practices for several decades now, especially for treating gastrointestinal diseases. CB supplementation has also been found to have an active effect across mouse models of colitis, antibiotic-associated diarrhea, ulcerative colitis, and irritable bowel syndrome (40–42). It can also prevent CRC to some extent. Several studies have shown that gut microbiota dysbiosis is related to CRC (43–45). Compared to healthy individuals, patients with CRC often suffer from a reduction in butyrate (46, 47). As a type of anaerobe with a major production of butyric acid, CB makes part of normal gut microbiota (23, 48). In addition, it also regulates immune cells and immune factors with respect to local intestinal immunity and consequently reverses the development of intestinal inflammation (49, 50). Butyric acid, as the metabolite of CB, has a major role in the intestine, such as maintaining the intestinal barrier functions, improving the intestinal microecological balance, providing energy, and regulating the functions of immune cells and intestinal motility through the enteric nerve (51).

The normal human intestine harbors various microbes, such as bacteria, viruses, fungi, and protozoa, amounting to a total number of approximately 1×10^14^, which is > 10-folds of the total number of cells in the human body (52, 53). Previous examinations have revealed > 1000 types of intestinal bacteria, most of which are obligate anaerobes, such as *Firmicutes*, *Bacteroidota*, *Proteobacteria*, and *Actinomycetes* (54). The microbe environment in the human intestine is complex. About 90% of normal gut microbiota in the human body are *Firmicutes* and *Bacteroides* at the phylum level (55). Several studies have reported that IBD patients have decreased gut microbiota (56–64). In the present study, we measured alpha diversity using the Simpson scale, which showed that most of the bacteria manifested similar tendencies. However, the control group harbored microbiota with a significantly higher alpha diversity than the supernatant group, which was inconsistent with the previous findings (31). Most studies reported a reduced number of *Firmicutes*, while the number of *Proteobacteria* was found to be increased in the intestine of IBD patients (56–58, 65, 66). At the phylum level, we found that the percentage of *Firmicutes* was higher in the bacteria group than in the control group. Most Firmicutes are beneficial bacteria that have critical roles in energy supply and development of the host’s intestinal epithelial cells. Recent studies on the association between abnormal alterations of gut microbiota and intestinal cancer have shown that the changes in gut microbiota structures have specific roles in the development and progression of CRC (67), while the tumor microenvironment enriches the pathogenic microbes and reduces the probiotics (68). This study showed that at the class level, the percentage of CB was the highest in the bacteria group. Moreover, differences were detected in the predominance of bacterial communities in these groups, with Firmicutes phylum being the most predominant bacteria in the supernatant group.

CB is an obligate anaerobe characterized by the production of butyric acid, which directly provides energy for colon cells and alleviates the symptoms of inflammation (69). In the mice model of diarrhea induced by gut microbiota alteration, CB regulates gut microbiota homeostasis, improves barrier functions and mucosal immune cell dysregulation, and promotes the restoration of homeostasis (70). The current findings showed that during the progression into CAC in AOM+DSS-induced mice, various manifestations, including diarrhea and hematochezia, repeatedly appeared in the mice. However, after treatment with CB and the supernatant, these manifestations of colon inflammation were significantly alleviated, and the body weight and DAI also rapidly recovered. In addition, the tumor sizes in the bacteria and supernatant groups were significantly smaller than in the control group, which could be attributed to the high levels of butyrate in the supernatant.

Previous studies have shown that CB activates the Th1 immune response, induces native T lymphocyte differentiation into regulatory T (Treg) cells and maturation, upregulates the expression of anti-inflammatory cytokine (IL-10) and promotes the immune tolerance of the system (71). IL-10 is a potent anti-inflammatory cytokine that is produced by almost all immune cells. In addition, tumor cells and tumor-invaded macrophages also secrete IL-10 (72). Cai et al. (73) used CB capsules to treat patients with ulcerative colitis (UC) and found that the serum levels of specific IgE, IL-4, and TNF-α were reduced; therefore, CB could improve UC. In addition, elevated NF-κB expression was detected in the colon’s epithelium mucosae and mucosal lamina propria. It also increased the expressions of various pro-inflammatory cytokines, such as IL-6, IL-1β, and TNF-α, which then worsened the inflammatory responses (74). TNF-α is a tumor-induction factor that induces the production of reactive oxygen species and promotes DNA damage, thus inducing the carcinogenicity of cell (75). In this study, the serum levels of IL-6, IL-10, and TNF-α were measured, and the findings showed that the levels of IL-6 and TNF-α were reduced, while IL-10 level was increased in both the bacteria and supernatant groups compared to the control group, indicating that both the CB culture and supernatant had anti-inflammatory effects.

NF-κB signaling pathway has a major role in the conversion processes of inflammation and tumors by stimulating the expression of pro-inflammatory cytokines and anti-apoptosis genes (76). An NF-κB signaling pathway is upregulated in the inflammatory intestine mucosae of mice models of UC and CAC (77). The administration of AOM and DSS activates IKK and the expression of anti-apoptosis protein Bcl-xL, while the deficiency of IκBβ in intestinal mucosal cells upregulates the expression of pro-apoptosis proteins, such as Bak and Bax, thus promoting cell apoptosis. These findings suggest that the NF-κB signaling pathway inhibits cell apoptosis to promote the progression of tumors (75, 76). Therefore, CB supernatant could also have high anti-tumor effects. qPCR and Western blot results showed that the levels of anti-apoptosis proteins NF-κb/p65 and Bcl-2 were significantly lower in the bacteria and supernatant groups compared to the control group, while the level of pro-apoptosis protein Bax level was higher in the bacteria and supernatant groups, which could be associated with the effects of CB culture and supernatant on reducing the expression of pro-inflammatory cytokines, such as IL-6 and TNF-α, and increasing the expression of IL-10. These findings could also be associated with the capability of CB culture and supernatant in regulating the NF-κb/p65 pathway to regulate the development and progression of CRC, which is consistent with previous studies.

## 5 Conclusion

Our findings suggest that CB and supernatant could at least partially inhibit the release of inflammatory mediators, protect the intestinal mucosal, and inhibit the development of colitis and CAC by regulating the NF-κB signaling pathway and gut microbiota. This may be due to the large amount of butyrate produced by CB and the large amount of butyrate in the supernatant. In addition, butyrate may have a key role in the development of colorectal cancer.It may partially inhibit the release of inflammatory mediators, protect the intestinal mucosal, and inhibit the development of colitis and CAC by regulating the NF-κB signaling pathway and gut microbiota.

Therefore, CB preparations such as butyrate-rich preparations may be considered a promising option for treating CRC.

## Data availability statement

The raw data supporting the conclusions of this article will be made available by the authors, without undue reservation.

## Ethics statement

The animal study was reviewed and approved by Ethics Committee of North Sichuan Medical College.

## Author contributions

ST supervised and organized the project. WP conducted the experiments, interpreted the data, and wrote the manuscript. HZ performed data curation and helped to behavioral experiments. TZ provided some resources. XG completed the pathological treatment and analysis of colon tissue. XW contributed to data collection and validation. All authors contributed to the article and approved the submitted version.
